# Six Newly Sequenced Chloroplast Genomes From Trentepohliales: The Inflated Genomes, Alternative Genetic Code and Dynamic Evolution

**DOI:** 10.3389/fpls.2021.780054

**Published:** 2021-12-08

**Authors:** Jiao Fang, Benwen Liu, Guoxiang Liu, Heroen Verbruggen, Huan Zhu

**Affiliations:** ^1^Key Laboratory of Algal Biology, Institute of Hydrobiology, Chinese Academy of Sciences, Wuhan, China; ^2^University of Chinese Academy of Sciences, Beijing, China; ^3^School of BioSciences, University of Melbourne, Melbourne, VIC, Australia; ^4^State Key Laboratory of Freshwater Ecology and Biotechnology, Institute of Hydrobiology, Chinese Academy of Sciences, Wuhan, China

**Keywords:** *Cephaleuros*, chloroplast genome, green algae, inverted repeats, terrestrial algae, Trentepohliales

## Abstract

*Cephaleuros* is often known as an algal pathogen with 19 taxonomically valid species, some of which are responsible for red rust and algal spot diseases in vascular plants. No chloroplast genomes have yet been reported in this genus, and the limited genetic information is an obstacle to understanding the evolution of this genus. In this study, we sequenced six new Trentepohliales chloroplast genomes, including four *Cephaleuros* and two *Trentepohlia*. The chloroplast genomes of Trentepohliales are large compared to most green algae, ranging from 216 to 408 kbp. They encode between 93 and 98 genes and have a GC content of 26–36%. All new chloroplast genomes were circular-mapping and lacked a quadripartite structure, in contrast to the previously sequenced *Trentepohlia odorata*, which does have an inverted repeat. The duplicated *trnD-GTC*, *petD*, and *atpA* genes in *C. karstenii* may be remnants of the IR region and shed light on its reduction. Chloroplast genes of Trentepohliales show elevated rates of evolution, strong rearrangement dynamics and several genes display an alternative genetic code with reassignment of the UGA/UAG codon presumably coding for arginine. Our results present the first whole chloroplast genome of the genus *Cephaleuros* and enrich the chloroplast genome resources of Trentepohliales.

## Introduction

The order Trentepohliales (Ulvophyceae, Chlorophyta) consists of approximately 80 species of terrestrial green algae that are widely distributed in tropical, subtropical and temperate regions with humid climates (Thompson and Wujek, 1997; Suto and Ohtani, 2009; Zhu et al., 2017). The species are found on a large variety of subaerial substrates including rocks, tree bark, leaves, tree trunks, building walls, wood and metal (Zhu et al., 2019). Algae in the Trentepohliales are characterized by uniseriate branched filaments, net-like chloroplasts without pyrenoids, zoosporangia supported by a special curved supporting cell, cell walls with plasmodesmata and cytokinesis by production of a phragmoplast similar to that of the vascular plants (Brooks et al., 2015). Many Trentepohliales have a striking color due to abundant carotenoids in their cells, facilitating resistance to the strong ultraviolet radiation in subaerial habitats (Liu et al., 2012) and offering prospects for uses in the fields of health care products, cosmetics and feed (Li et al., 2014). Some species of the order Trentepohliales live in association with fungi, forming lichens (Nelsen et al., 2011; Kosecka et al., 2020). Several species of the genus *Cephaleuros* Kunze ex Fries are well-known plant pathogens, including *C. virescens* and *C. parasiticus*. The infections caused by them are often referred to as ‘red rust’ or ‘algal spot disease,’ which can damage economically important crops (e.g., tea, coffee, citrus) (Wolf, 1930; Nelson, 2008; Brooks et al., 2015). The treatments for algal spot disease has always been a headache for phytopathologists (Browne et al., 2019; Lee et al., 2020).

The relationships among the major lineages of core Chlorophyta have been evaluated based on nuclear and plastid datasets as well as genome-scale data derived from chloroplast genomes or transcriptomes (Cocquyt et al., 2010; Fučíková et al., 2014; Fang et al., 2018; Li et al., 2021). However, some critical data is missing for the Ulvophyceae, with the scarcity of complete chloroplast genomes in Dasycladales and Trentepohliales being particularly problematic. Previous studies of Trentepohliales have focused mainly on morphological traits, and phylogenies based on traditional molecular markers such 18S rDNA, ITS and *rbc*L often lacked the resolution that could be provided by chloroplast genomes (López-Bautista et al., 2006; Rindi et al., 2009; Zhu et al., 2017).

Chloroplast genomes have become a work horse for phylogenetic and evolutionary studies on algae and higher plants with its unique advantage of matrilineal inheritance (Sun et al., 2016; Xu et al., 2021) and variable rates of molecular evolution across regions (Costa et al., 2016). High-throughput sequencing has facilitated a dramatic increase of green algal chloroplast genome sequencing, but so far, only a single complete chloroplast genome has been published in the order Trentepohliales (Zhu et al., 2019), and none in the genus *Cephaleuros*.

The goal of this study is to address this shortfall in chloroplast genome information in the Trentepohliales by sequencing and analyzing six chloroplast genomes including four in the genus *Cephaleuros*. We provide a detailed comparative account of genome features within the order and with other lineages of the Ulvophyceae.

## Materials and Methods

### Samples Collection and Culture Conditions

Strains BN17, YN1242, and YN1317 were collected in Xishuangbanna Tropical Botanical Garden (Yunnan Province, China), strains GD1942, GD1927 in South China Botanical Garden (Guandong Province, China) and strain SAG 42.85 was purchased from University of Göttingen, Germany (SAG^1^). Detailed specimen information was presented in Supplementary Table S1. All strains were grown at the Freshwater Algal Herbarium (HBI), Institute of Hydrobiology, Chinese Academy of Sciences, Wuhan, China. They were cultivated in BBM medium (Bischoff and Bold, 1963), maintained in a culture chamber at a temperature of 20–25°C and illumination at 35–50 μmol photons m^–2^ s^–1^ for a 12 h:12 h light:dark cycle.

### Genome Sequencing, Assembly, and Annotation

The sequencing library was prepared using NEBNex Ultra DNA Library Prep Kit for Illumina (New England Biolabs, Ipswich, MA, United States), and sequenced on the Illumina NovaSeq 6000 platform by Benagen company (Beijing, China). The raw data is trimmed with SOAPnuke v. 1.3.0 (Chen et al., 2018). Sequencing data were assembled with SPAdes v. 3.13.0 (Bankevich et al., 2012). Chloroplast genes were annotated using PGA (Qu et al., 2019) and MFannot^2^. The annotation results of protein-coding genes were further polished using Blast search (Altschul et al., 1997) with homologous genes from closely related chloroplast sequences. The tRNA and rRNA genes were identified using tRNAscan-SE v 1.23 and RNAmmer, respectively (Lowe and Eddy, 1997; Karin et al., 2007). All open reading frames (ORFs) (with length > 300 bp) were extracted by ORFfinder^3^. Intron boundaries were determined by comparing intron-containing genes with homologs without introns. In order to identify the intron class (group I or group II), RNA secondary structures of the introns were predicted using RNAweasel (Michel et al., 1989; Michel and Westhof, 1990). The circular chloroplast genome maps were drawn using OrganellarGenomeDRAW v 1.3.1 (Stephan et al., 2019). Annotated sequences were deposited in the NCBI GenBank database under the accession numbers listed in Table 1. The relative amino acid frequencies at premature termination UGA or UAG position was performed in web WebLogo v 2.8.2 (Crooks et al., 2004). The frequency of the six canonical and two non-canonical arginine codons was calculated for each species using MEGA v 4.0 (Tamura et al., 2007). The distribution of codon frequencies was obtained using the ggplot2 package in R v 3.6.0 (R Core Team, 2016).

**TABLE 1:** Summary of chloroplast genome features in Trentepohliales.

Species	Strains	GenBank Number	Size (bp)	GC content (%)	^a^Total Genes	Protein coding Genes	CDS (plus/minus)		tRNA	rRNA	^b^ORFs	Quadripartite Structure
							±	−				
Cephaleuros virescens	SAG 42.85	MW822747	314936	36.1	95	64	20	44	28	3	96	No
Cephaleuros tumidae-setae	BN 17	MW822748	282795	33.2	95	64	37	27	28	3	50	No
Cephaleuros karstenni	GD1942	MZ334628	371192	29.9	98	65	40	25	30	3	39	No
Cephaleuros parasiticus	GD1927	MZ334627	266729	35.9	96	64	24	40	29	3	49	No
Trentepohlia sp.	YN1242	MZ334625	216308	25.9	93	65	30	35	25	3	16	No
Trentepohlia sp.	YN1317	MZ334626	408697	31.7	94	65	33	42	26	3	57	No
Trentepohlia odorata	DZ1317	MK580484	399372	29.8	97	63	19	44	30	3	95	Yes

*^a^A sum of protein-coding genes, tRNA genes, rRNA genes.*

*^b^ORFs length > 100 aa.*

### Comparative Genomics

Synteny comparison was visualized using the progressiveMauve program under Mauve v 2.3.1 (Darling et al., 2010) with default settings after adjusting all genomes to start at the 16S rRNA gene. Only one copy of the IR region in *T. odorata* was included in the analysis.

### Phylogenetic Analyses

The first phylogenetic analyze is based on 18S rDNA to verify the identity of our strains and place them among known Trentepohliales biodiversity. The next analysis used concatenated data of 31 chloroplast genes from chloroplast genomes sampled across the Chlorophyta, in order to study molecular evolution of the chloroplast genomes in a broader taxonomic context.

The 18S rDNA of strains BN17, GD1942, and GD1927 were obtained as described in Fang et al. (2021) and sequences for strains SAG 42.85, YN1242, YN1317 and a range of other Trentepohliales were downloaded from NCBI^4^. Sequences were aligned with MAFFT 7.0 (Katoh and Standley, 2014) and ambiguous regions removed using trimAl 1.2 (Capella-Gutierrez et al., 2009). We performed Maximum Likelihood (ML) analysis using the IQ-TREE web server^5^ (Trifinopoulos et al., 2016) and Bayesian Inference (BI) in MrBayes v 3.1.2 (Huelsenbeck and Ronquist, 2001). For ML, the K3P + I + G4 model was selected using IQ-TREE as the best-fit model according to the Bayesian information criterion (BIC), and 1,000 bootstrap replicates were used to estimate statistical reliability. For BI, ModelFinder program of PhyloSuite v 1.2.2 was used to select the best-fitting model (Kalyaanamoorthy et al., 2017) available among those implemented in MrBayes, in this case GTR + I + G. Markov chain Monte Carlo analyses were run with four Markov chains for 1,000,000 generations or more, with trees sampled every 1,000 generations. The first 25% of the calculated trees were discarded as burn-in, and the remaining samples were used to construct a Bayesian consensus tree with posterior probabilities. A stationary distribution was assumed when the average standard deviation of the split frequencies was lower than 0.01.

For phylogenetic analysis based on chloroplast genomes, 31 conserved protein-coding genes widely present among green algae were used (Zhu et al., 2019): *atpA*, *atpB*, *atpF*, *atpH*, *clpP*, *petB*, *petD*, *petG*, *psaB*, *psaC*, *psbA*, *psbB*, *psbJ*, *psbK*, *psbM*, *psbN*, *psbZ*, *rbcL*, *rpl14*, *rpl16*, *rpl20*, *rpl2*, *rpl5*, *rps11*, *rps18*, *rps19*, *rps7*, *rps8*, *rps9*, *tufA*, *ycf3*. Prasinophytes were used as outgroup taxa. The GenBank accession numbers used to reconstruct phylogenetic inference in core Chlorophyta are listed in Supplementary Table S2. PhyloSuite v 1.2.2 was used to prepare data for analysis (Zhang et al., 2020). The 31 gene alignments were produced with MAFFT (Katoh and Standley, 2014) using codon alignment mode. Ambiguously aligned fragments were removed using Gblocks with the following parameter settings: minimum number of sequences for a conserved/flank position (45/45), maximum number of contiguous non-conserved positions (8), minimum length of a block (10), allowed gap positions (with half) (Talavera and Castresana, 2007). A total of 18543 positions were retained for the phylogenetic inference on nucleotide data set. Data concatenation used the “Concatenate Sequence” program of PhyloSuite v 1.2.2 (Zhang et al., 2020). ModelFinder was used to select the best-fit partition model using the BIC (Kalyaanamoorthy et al., 2017). The partitions and best-fit models are shown in Supplementary Table S3. ML analyses were carried out using IQ-TREE (Trifinopoulos et al., 2016). The constructed phylogenetic tree was visualized using the software Figtree v 1.4.4^6^.

## Results

### Chloroplast Genome Features

**FIGURE 1:**
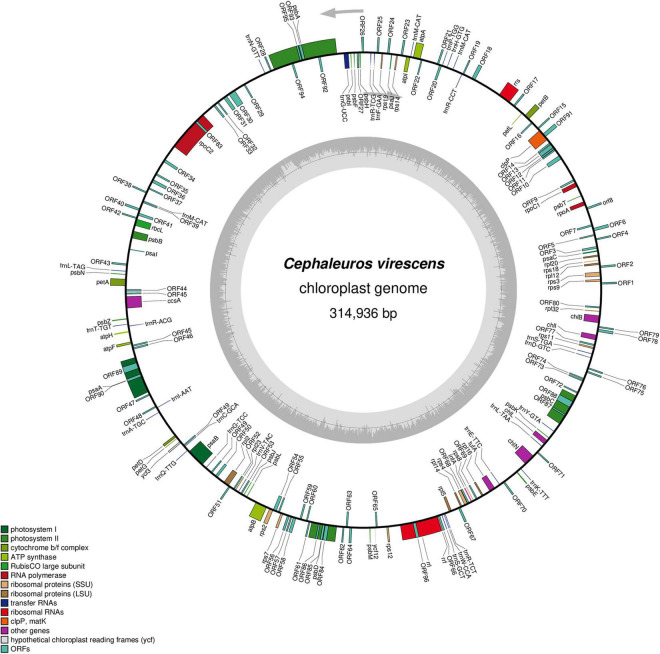
Gene map of the *Cephaleuros virescens* chloroplast genome. The gray circle on the inside shows a graph of the GC content. Arrows show the direction of transcription. Genes are color-coded according to the functional categories listed in the legend at the bottom left.

We sequenced chloroplast genomes from two *Trentepohlia* and four *Cephaleuros* strains (Figure 1 and Supplementary Figure S1). The annotated chloroplast genomes were submitted to GenBank under the accession numbers given in Table 1. An 18S rDNA analysis situated the sequenced strains in the broader Trentepohliales biodiversity, showing well-resolved positions for *Cephaleuros tumidae*-*setae* BN 17 and *Cephaleuros karstenii* GD1942. *Cephaleuros virescens* was paraphyletic, with *Cephaleuros virescens* SAG 42.85 being closely related to *Cephaleuros parasiticus* GD1927 (Supplementary Figure S2). The positions of *Trentepohlia* sp. YN1317 and *Trentepohlia* sp. YN1242 received weak support values in both ML and BI analyses. The result also indicated that *Trentepohlia odorata* was most closely related to *Trentepohlia annulata* and *Trentepohlia* cf. *umbrina* (Supplementary Figure S2), consistent with previous work (Zhu et al., 2019).

Trentepohliales chloroplast genomes ranged in size from 216,308 to 408,697 bp. All the newly sequenced chloroplast genomes of Trentepohliales were circular-mapping and lacked the quadripartite structure (Figure 1 and Supplementary Figure S1) seen in many other Chlorophyta including *Trentepohlia odorata*. GC content was low and showed minor differences between species, ranging from 25.9% in *Trentepohlia* sp. YN1242 to 36.1% in *Cephaleuros virescens* SAG 42.85. The distribution of protein-coding genes in the double strands was skewed and varied among species. The distribution of protein-coding genes of *Trentepohlia odorata* was the most uneven (±, 19/44). The free-standing ORFs of length > 100 aa detected in the genomes varied between 16 and 95, with *Trentepohlia odorata* having the most (95), followed by *Cephaleuros virescens* (82).

### Gene Content

The 6 completely sequenced cpDNAs contain 93-98 genes, shared nearly identical gene repertoires (Supplementary Table S4) except for a handful of genes. For example, *rpl12*, *rpl32*, *petL* were not found in *T. odorata*, but were present in the rest of the Trentepohliales taxa. The *ycf12* gene was lost in *Trentepohlia odorata*, *Cephaleuros parasiticus*, and *Cephaleuros karstenii*, *ycf1* was lost in *Cephaleuros tumidae*-*setae* and *Cephaleuros virescens*.

There were also some duplicated genes, including the *clpP* gene with two copies in *T. odorata*. The *trnE-TTC*, *trnD-GTC*, *petD*, and *atpA* genes have two copies in *Cephaleuros karstenii* and *trnG-TCC* has two copies in *Cephaleuros virescens* and *Cephaleuros parasiticus*.

The gene repertoire of the chloroplast genome was quite homogeneous in Trentepohliales and similar to other Ulvophyceae members. A total of 77 genes including two ribosomal RNAs and 21 transfer RNAs are shared by all members of Ulvophyceae (Supplementary Table S4). In comparison with other lineages of Ulvophyceae, however, some chloroplast protein coding genes were lost in Trentepohliales, but found in other Ulvophyceae chloroplast genomes, such as *ycf20*, *ycf4*, *rpl19*, *rpl36*, *psaM*, *cemA*, *ftsH*, and *accD* genes. The genes that were lost in Trentepohliales have diverse functions including inorganic carbon dioxide uptake into chloroplasts (*cemA*), photosynthesis (*ycf4*), translation (*rpl1*2, *rpl32* lost in *T*. *odorata*), and proteins of unknown function (*ycf20*). Two tRNAs (*trnI-AAT* and *trnR-TCG*) were found in Trentepohliales but were missing in all other analyzed Ulvophyceae and the *trnI-GAT* gene was found in all Ulvophyceae except the Trentepohliales (Supplementary Table S4).

### The Alternative Genetic Code

**FIGURE 2:**
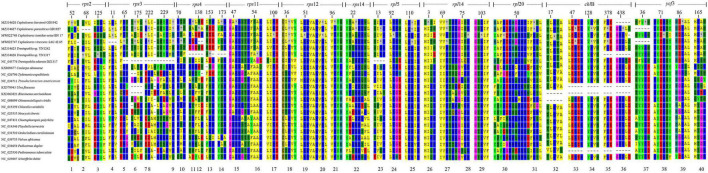
The amino acid sequence alignment for seven chloroplast genes of Trentepohliales and other representatives of Chlorophyta. Positions corresponding to UGA/UAG codons in Trentepohliales are indicated by an asterisk. The vertical blank space between alignment matrix represents regions of the sequence alignment that were omitted for simplicity. For each gene, position in the alignment is indicated by the numbers shown above the sequence.

**FIGURE 3:**
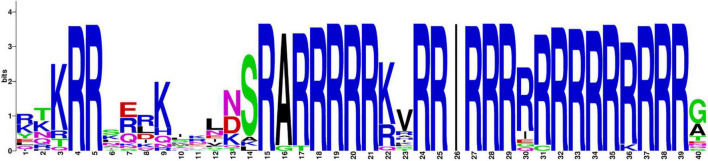
Sequence logo of the position weight matrix reporting the relative amino acid frequencies in the alignment for each abnormal UGA/UGA position in Trentepohliales.

For the newly sequenced Trentepohliales chloroplast genomes, all three codons (UAA/UAG/UGA) used as a genuine termination codon present in plastid genes in Trentepohliales. Among them, UAA stop codons are common. However, some apparent UGA and UAG codons were also found in the otherwise well-conserved CDSs of several protein-coding genes, suggestive of an alternative genetic code (Figure 2 and Supplementary Table S5). The genes with in-frame UGA/UAG codons included ribosomal proteins (*rps* and *rpl*) and photosynthesis-related genes (*chlB* and *ycf3*). At 20 of these 40 positions, the corresponding amino acid residue in orthologous genes was highly conserved (i.e., present in almost all taxa in the alignment matrix), and most in-frame UAG/UGA codons were found at highly conserved positions where other green algae encode arginine (Figure 3). These results suggest that the most in-frame UGA/UAG codons may be reassigned as arginine. Additionally, the UAG codons of the third position of the *rpl14* gene were found at highly conserved positions where other green algae encode isoleucine (Figure 3). UAG/UGA codons at conserved arginine and isoleucine positions, in combination with conservation of the sequence before and after the in-frame UAG/UGA codon, further support the presence of alternative genetic code. Several different amino acids were also observed at these positions: 52th and 67th in *rps2*, and 46th in *rpl20* (Figure 2), the amino acid in these positions were not conserved, and therefore the amino acid coded by the UGA/UAG codon could not be determined with certainty.

**FIGURE 4:**
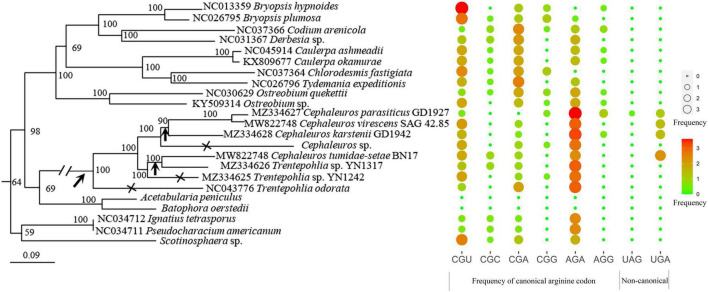
The estimated frequencies of arginine codon usage. Canonical codon AGA is most commonly used, followed by other canonical codon CGU. Among the non-canonical codons, UGA is used more commonly then UAG. (1) A single origin of the non-canonical code along the branch leading to *C. parasiticus*, *C. virescens*, *C. karstenii*, *C. tumidae-setae*, and *Trentepohlia* sp. YN1317, and a subsequent reversal to the standard code in *Cephaleuros* sp., *Trentepohlia* sp. YN1242, and *Trentepohlia odorata* (indicated with oblique arrow and cross). (2) A stepwise process of evolution of the non-canonical code with a single initiation of the process along the branch leading to *C. parasiticus*, *C. virescens*, *C. karstenii*, *C. tumidae-setae*, and *Trentepohlia* sp. YN1317, followed by a completion of the process in all species except *Cephaleuros* sp., *Trentepohlia* sp. YN1242 and *Trentepohlia odorata* (oblique arrow combined with vertical arrow). (3) Two independent gains of the non-canonical code in the Trentepohliales (vertical arrow).

The estimated evolution of arginine codon usage frequencies was shown in Figure 4 (only the portion of Ulvophyceae taxon were shown). In general, the canonical codon AGA is most commonly used, followed by the canonical codon CGU. Among non-canonical arginine codons, UGA being more common than UAG codon. The bias toward AGA and UGA is likely a product of the overall bias of these genes for GC residues, leading to increased use of G in the second codon position.

### Introns

All analyzed Trentepohliales chloroplast genomes contained introns, and the distribution and types of introns are listed in Supplementary Table S5. The number of introns ranged from 16 (*Trentepohlia* sp. YN1242) to 62 (*C. tumidae*-*setae* BN 17). Among the newly sequenced Trentepohliales chloroplast genomes, only *psbC*, *psbA*, and *rrl* genes always contained introns. Intron prevalence was not necessarily correlated with genome size; for example even though the *Cephaleuros tumidae*-*setae* chloroplast genome was not the largest in Trentepohliales, it had the largest number of introns (Supplementary Table S6).

The *atpA* gene in *Cephaleuros parasiticus* contains one introns, it identified as a group I intron, which is commonly found in this gene in other green algae (Pombert et al., 2005). *PsbA* gene in *Trentepohlia* sp. YN1317 has six introns, that five introns were identified as group II introns, one as group I intron. The third and fourth introns contain one and three ORFs respectively. The *psbA* gene in *Tydemania expeditionis* had also been found two ORFs (Leliaert and Lopez-Bautista, 2015). Introns are common in *psbC* genes of Trentepohliales. The *psbC* of *C. parasiticus* and *Trentepohlia* sp. YN1242 both contain an intron, both of which are group I introns. The *psbC* introns in remaining species contain both group I and group II introns. The intron in *psbD* gene were present in all newly sequenced chloroplast genomes of Trentepohliales, and also found in *Caulerpa okamurae* and *Tupiella akineta* (Zheng et al., 2020).

### Synteny Analysis

**FIGURE 5:**
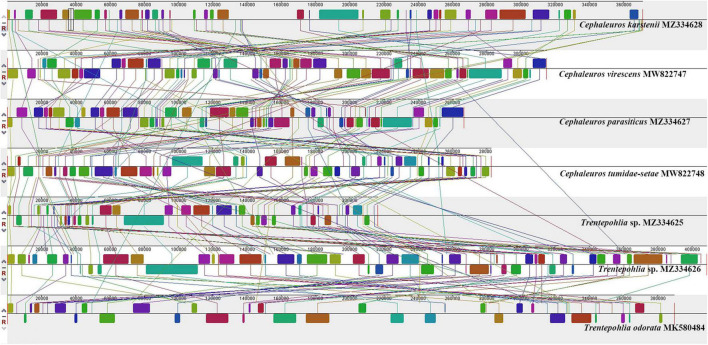
Synteny comparison of seven chloroplast genomes in the order Trentepohliales using progressiveMauve.

ProgressiveMauve alignment of seven Trentepohliales chloroplast genomes showed fragmentation of the genomes into many small locally collinear blocks (LCBs) and suggested high levels of rearrangements across the Trentepohliales green algae (Figure 5). A separate alignment within *Cephaleuros* identified > 20 LCBs and considerable rearrangements and inversions (Supplementary Figure S3).

**FIGURE 6:**
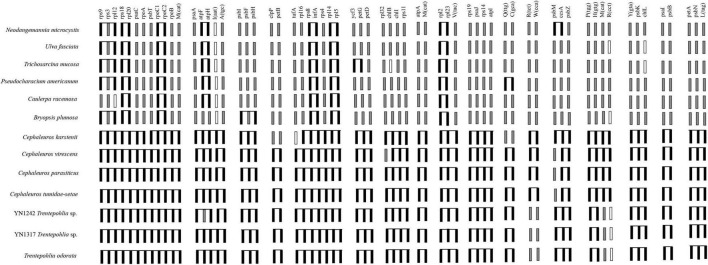
Comparison of gene clusters conserved in Trentepohliales with gene order found in six Ulvophycean lineages. Black connected boxes indicate gene clusters that made up of contiguous genes. Black boxes that are contiguous but are unlinked indicate that the corresponding genes are not adjacent on the genome. Gray boxes indicate genes that are located elsewhere on genome. White boxes indicate genes that are missing from the chloroplast genomes.

Across the seven Trentepohliales chloroplast genomes, 17 conserved gene clusters encoding 64 genes were found (Figure 6). Nine of these gene clusters were partially conserved, and the rest were intact across all genomes. The incomplete clusters (*trnR-TCT–trnW-CCA*, *trnP-TGG–trnH-GTG–trnM-CAT–trnR-CCT*, *psbM*-*ccsA*-*psbZ*) differed between the genera *Cephaleuros* and *Trentepohlia*. Despite the reported close relationship between *C. virescens* and *C. karstenii* (Fang et al., 2021), 7 out of 17 gene clusters between them had differences. The breakage of two gene clusters (*trnQ-TTG–trnC-GCA*, *clpP–petB*) in *C*. *karstenii* set it apart from all other investigated species of the order Trentepohliales (Figure 6).

The few clusters that are conserved in other Ulvophyceae green algal (based on (Turmel et al., 2009) were also conserved among Trentepohliales, albeit some only partially (Supplementary Figure S4). It is worth noting that the six gene clusters, *rps12*-*rps7*, *rps2*-*atpI*-*atpH*-*atpF*-*atpA*, *psbB*-*psbT*, *petB*-*petD*, *atpB*-*atpE*, and *psbD*-*psbC* were all conserved in other Ulvophyceae, but not or only partially in Trentepohliales. On the other hand, some gene clusters consistently present in the Trentepohliales (e.g., *rps19*-*psaJ*-*rps14*-*atpI*, *petA*-*psbN*-*trnL*-*TAG*) are not present among the six other Ulvophyceae in the analysis (Supplementary Figure S4). Finally, Trentepohliales and Bryopsidales shared the *petA*-*petL*-*petG* gene cluster (Supplementary Figure S4).

### Comparison of Chloroplast Genomes in Ulvophyceae

**FIGURE 7:**
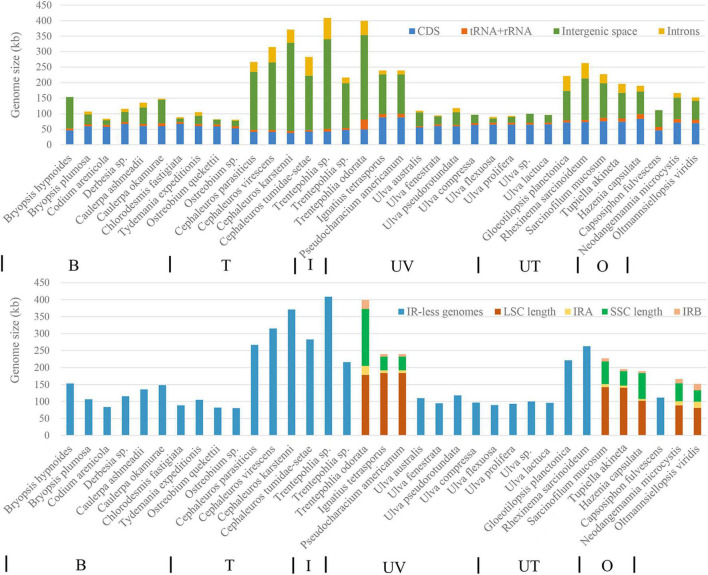
Contribution of different features to the chloroplast genomes compared in this study. The **top panel** shows contributions of CDS, intronic, intergenic, and tRNA + rRNA. Note that CDS regions do not include hypothetical open reading frames (ORFs). The **bottom panel** shows sizes of the SSC, IR, and LSC regions. Chloroplast genomes lacking the IR are represented in blue. From left to right at the bottom of picture, B, T, I, UV, UT, and O represent the order Bryopsidales, Trentepohliales, Ignatiales, Ulvales, Ulotrichales, and Oltmannsiellopsidales.

Among the 35 chloroplast genomes of Ulvophyceae included in the phylogenetic tree, only eight possessed an inverted repeat region (Figure 7). Among these IR-containing chloroplast genomes, *Trentepohlia odorata* has the largest chloroplast genome due to the very long single-copy (SC) and inverted repeat regions. IR-less chloroplast genomes are mainly found in Bryopsidales, Trentepohliales, and Ulvales. In the seven Trentepohliales chloroplast genomes, the length of CDS region was almost equal, and the difference in chloroplast genome size was largely caused by length of intergenic regions and intron content (Figure 7), consistent with findings in other taxa (Turmel et al., 2017). Likewise, the greatest influence on chloroplast genome size among Ulvophyceae more broadly was the length of intergenic regions, followed by intron content and the length of coding regions. Remarkably, the Trentepohliales chloroplast genomes were the largest among the 35 surveyed Ulvophyceae, with six out of seven Trentepohliales chloroplast genomes exceeding 250 kb (Figure 7).

**FIGURE 8:**
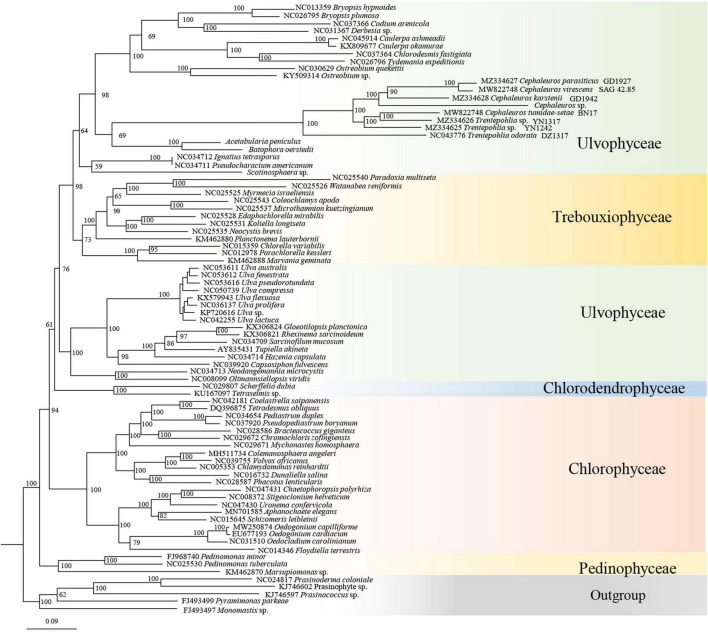
ML phylogenetic tree inferred from nucleotide dataset partitioned by gene position. Maximum likelihood bootstrap values (1000 replicates) are given near the nodes. Scale bar indicates substitutions per site.

**FIGURE 9:**
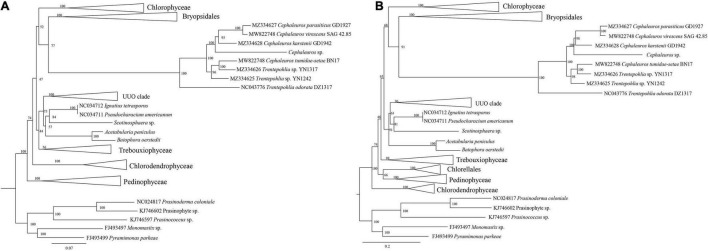
ML phylogenetic tree of the Chlorophyta. **(A)** ML phylogenetic tree from the nucleotide (nt) alignment (first two codon positions) of 31 concatenated chloroplast genes. Node support is given as maximum-likelihood (ML) bootstrap values. **(B)** ML phylogenetic tree of the Chlorophyta constructed by using concatenated amino acid (aa) data. Maximum likelihood bootstrap values (1000 replicates) are given near the nodes.

Trentepohliales were recovered as the sister lineage to Dasycladales in ML phylogenetic trees constructed by nucleotide (nt) data with two methods (partitioned by gene position, and codon position) (Figure 8 and Supplementary Figure S5), but support values for this relationship are unconvincing (Support value: 69 in Figure 8, 62 in Supplementary Figure S5). Taking into account the nucleotide composition bias that mainly affected by the position of the third codon, a phylogenetic tree inferred from the nucleotide alignment without third codon positions was summarized in Figure 9A. This result indicated that Trentepohliales was more closely related to Bryopsidales with bootstrap values 77, the support value in phylogenetic tree based on the amino acid (aa) data set were higher (Figure 9B). No matter what type of phylogenetic tree, the internal relationships of Trentepohliales were constant, and non-monophyly of the genus *Trentepohlia* strongly supported by plastid phylogenomics. An interesting observation was that the Trentepohliales have substantially longer branch lengths than other Chlorophyta lineages, indicating an elevated rate of substitution in the group. In addition, the Ulvophycean were polyphyletic in all the above phylogenetic analyses, which is consistent with consistent with previous work (Sun et al., 2016; Fang et al., 2018; Zhu et al., 2019).

## Discussion

### Inflated Chloroplast Genomes in Terrestrial Algae

The newly presented chloroplast genomes clearly establish the Trentepohliales as having very large chloroplast genomes (216–408 kbp), much larger than most published Chlorophyta chloroplast genomes. This difference in chloroplast genome size results primarily from variation in non-coding regions including the length of intergenic space and introns (Figure 7). We note that other terrestrial algae also have large chloroplast genomes, including *Floydiella terrestris* (521,168 bp) in the Chaetopeltidales (Brouard et al., 2010) and *Gloeotilopsis sarcinoidea* (262,888 bp) in the Ulotrichales (Turmel et al., 2015), and it is interesting to contemplate the possibility that there may be a common cause behind this, which we will expand upon below.

### Vanishing Inverted Repeats

Although quadripartite architecture is believed to be ancestral in the green algae (Civán̆ et al., 2014), the six new Trentepohliales chloroplast genomes have no inverted repeats, similar to the Bryopsidales (Leliaert and Lopez-Bautista, 2015; Cremen et al., 2018). In addition to Ulvophyceae, it was reported IR had been lost many times in other lineages of Chlorophyta, including at least twice in the Chlorophyceae (Brouard et al., 2010) and at least seven times in the Trebouxiophyceae (Turmel et al., 2015). Three possible models have been proposed to explain how the IR is lost (Turmel and Lemieux, 2017; Turmel et al., 2017), all of which seem to match well for some groups of organisms but not others, suggesting there are many paths toward losing an IR.

Chloroplast genomes of *Cephaleuros* species lack the IR region but *C. karstenii* features duplicated copies of some genes that we speculate may be remnants of the IR region. The IR-less *C. karstenii* genome can best be compared to that of IR-containing *T. odorata*, as they are similar in many ways. Our reasoning for a remnant IR lies in the *trnD-GTC*, *petD* and *atpA* genes, which are duplicated in *C. karstenii* and located near the IR/LSC boundary in *T. odorata*. Adding to the evidence is that the two copies of *petD* and *atpA* in *C. karstenii* have the same length but are oriented in opposite directions, a characteristic shared with the IR region. It is interesting to note that *Oltmannsiellopsis viridis* has the *petD* gene in its IR regions. While this species is not a close relative, it suggests that *petD* might have been part of an ancestral IR and that this duplication has been maintained in the evolutionary lineage to *C. karstenii*. The two *trnD-GTC* sequences in the IR-less genome of *C. karstenii* could be the product of a duplication event or an IR remnant. The *trnD-GTC* gene is also duplicated in the IR-containing plastid of some Trebouxiophyceae, for example in *Dicloster acuatus* (Lemieux et al., 2014).

Remarkably, the two newly sequenced *Trentepohlia* chloroplast genomes lack IR regions even though *Trentepohlia odorata* does have quadripartite structure. This situation is well explained by observing the phylogenetic positions of these two newly sequenced *Trentepohlia* strains and *Trentepohlia odorata* based on the 18S rDNA phylogenetic tree (Zhu et al., 2017). The two newly sequenced *Trentepohlia* strains are both in the core *Trentepohlia* clade, sister to the *Stomatochroon* and *Cephaleuros* clades (Supplementary Figure S2; Zhu et al., 2017). *Trentepohlia odorata* on the other hand was located elsewhere in the phylogeny as sister to the core *Phycopeltis* group. Both newly sequenced *Trentepohlia* strains are thus more closely related to *Cephaleuros* than to *Trentepohlia odorata*. At present, only *Trentepohlia odorata* presents quadripartite structure, but it seems likely that with further sampling, additional strains with this structure would be recovered in the Trentepohliales.

### The Uncertain Phylogenetic Position of the Trentepohliales

Our phylogenetic analysis based on chloroplast genomes indicated that Trentepohliales are most closely related to Bryopsidales. While this is not consistent with some previous studies based on chloroplast genomes (Fučíková et al., 2014; Melton et al., 2015; Fang et al., 2018; Zhu et al., 2019), support for the sister relationship between Trentepohliales and Dasycladales is low in our phylogeny trees generated with the concatenated nucleotide (nt) data set treated with two methods (partitioned by gene position, codon position) (Figure 8 and Supplementary Figure S5). The close relationship relationship between Trentepohliales and Bryopsidales is better supported in phylogenetic trees based on amino acid datasets than nucleotide datasets (Figure 9). The in-depth phylogenetic work based on chloroplast data has shown that the relationships in the core Chlorophyta, particularly Bryopsidales, Dasycladales and Trentepohliales depend on analysis settings and whether data are analyzed at the nucleotide or amino-acid level (Fang et al., 2018; Jackson et al., 2018).

More gene-rich analyses based on nuclear genes show that the phylogenetic configuration of these orders is different from that obtained with chloroplast genome data, with Bryopsidales more closely allied with Chlorophyceae than with Dasycladales and Trentepohliales (Del Cortona et al., 2020; Gulbrandsen et al., 2021; Li et al., 2021). In these analyses of nuclear genes, Trentepohliales are recovered as sister to Cladophorales, an order that was not included in the present analysis because of their highly deviant chloroplast genomes divided into dozens of small hairpin chromosomes (Del Cortona et al., 2017).

### Highly Dynamic Trentepohliales Chloroplast Genomes

Part of the difficulty in resolving the relationships of Trentepohliales with other Ulvophyceae based on chloroplast genomes may lie in their elevated rates of substitution. This can clearly be observed from the long branches for Trentepohliales in all our phylogeny being inferred from a set of highly conserved genes with critical functions in chloroplast functioning. Such elevated rates of molecular evolution can have a range of causes including elevated rates of mutation, lower effective population sizes leading to elevated drift thereby fixing more mutations, or poorer DNA repair mechanisms (Lynch, 2007; Smith, 2016). It is impossible to determine from the data at hand what drives the elevated rates in Trentepohliales, but we can make conjectures based on the biology of the organisms. First, it can be argued that due to their subaerial-terrestrial habitat, Trentepohliales may experience higher levels of UV irradiation, which could lead to higher mutation rates. Second, compared to their aquatic counterparts, subaerial algae may experience poorer dispersal, leading to more spatial isolation and hence smaller effective population size. Both these factors, along with a range of other influences, could contribute to the observed higher rates of molecular evolution.

The cause for the expansion of chloroplast genomes in Trentepohliales is similarly difficult to pin down. The mutational hazard hypothesis (Lynch et al., 2006) suggests that genomes tend to inflate when mutation rates and effective population sizes are low. This seems at least partially incompatible with the high rates of molecular evolution in Trentepohliales, which may point toward a higher mutation rate, but as indicated above, the potentially small effective population sizes may contribute to elevated rates of evolution too. Inaccurate DNA repair mechanisms are another potential mechanism that can lead to expansion of non-coding parts of organelle genomes (Christensen, 2014) and could help explain the growth of genomes even when mutation rates are high (Smith, 2016). The proliferation of introns also contributes and certainly seems to be among the factors contributing to large Trentepohliales chloroplast genome sizes, not unlike the situation in the recently characterized heavily expanded mitochondrial genomes of Bryopsidales (Repetti et al., 2020).

The dynamic nature of Trentepohliales chloroplast genomes also extends to patterns of rearrangement, with low levels of synteny across the order (Figure 5) or even within single genera (Supplementary Figure S3). *Trentepohlia* and *Cephaleuros* differ in the conservation of gene clusters, for example, *trnR-TCT–trnW-CCA* (Figure 6), and some gene clusters that are conserved across other Ulvophyceae were broken up in the Trentepohliales (Supplementary Figure S4). High variability in chloroplast genomes architecture has also been observed among the Bryopsidales, which also lacks the quadripartite structure (Cremen et al., 2018), but this is by no means a universal association as some higher-level groups of algae without quadripartite structure have exceptionally well-preserved synteny, for example the Ostreobineae (Verbruggen et al., 2017; Pasella et al., 2021) and Nemaliales (Costa et al., 2016).

### Alternative Genetic Code

Deviations from the standard genetic code are universally present in mitochondrial genomes, chloroplast genomes and nuclear genes, and include reassignment of stop codons, loss of start and stop codons in some groups, including apicomplexan, dinoflagellate, green algae and ciliates (Hanyu et al., 1986; Lang-Unnasch and Aiello, 1999; Cocquyt et al., 2010; Matsumoto et al., 2011). In the current study, plastid genes in Trentepohliales have canonical stop codon (UAA/UGA/UAG) at the end, also have in-frame UGA/UAG codons. The reassigned UGA/UAG codons were shown to occur in 11 chloroplast genes of Trentepohliales (Supplementary Table S5). Based on the alignments, the most conserved positions where these codons occur encode arginine (R) in other green algae, suggesting this is the most likely amino acid encoded by this reassigned codon (Figure 3). Most codon reassignments can be attributed to changes in tRNAs, either by base modification, or RNA editing. In Trentepohliales, several in-frame UAG/UGA codons were found at highly conserved positions where other green algae encode arginine. Additionally, the alignment sequence before and after the in-frame UAG/UGA codons are also conservative, the possibility of base insertion and loss in RNA editing is low. Our results reveal the distribution of a non-canonical genetic code in the Trentepohliales, where arginine is encoded by canonical codons as well as non-canonical UAG and UGA codons. Surprisingly, non-canonical code in *Trentepohlia* sp. YN1242 and *Trentepohlia odorata* is not observed. More specifically, the Trentepohliales lineages with the non-canonical code form a paraphyletic group. Several models have been proposed to explain the origin of the genetic code, for example, a stepwise acquisition model, the ambiguous intermediate model, multiple independent gains of the non-canonical code (Cocquyt et al., 2010; Li et al., 2021). If the internal relationship of the Trentepohliales in phylogenetic tree is correct, we infer alternative evolutionary scenarios to explain the distribution of the non-canonical code on that tree: (1) a single origin of the non-canonical code along the branch leading to *C. parasiticus*, *C. virescens*, *C. karstenii*, *C. tumidae*-*setae*, and *Trentepohlia* sp. YN1317, and a subsequent reversal to the standard code in *Cephaleuros* sp., *Trentepohlia* sp. YN1242, and *Trentepohlia odorata* (Figure 4: indicated with oblique arrow and cross). (2) based on the ambiguous intermediate model, we infer that a stepwise process of evolution of the non-canonical code with a single initiation of the process along the branch leading to *C. parasiticus*, *C. virescens*, *C. karstenii*, *C. tumidae-setae*, and *Trentepohlia* sp. YN1317, followed by a completion of the process in all species except *Cephaleuros* sp., *Trentepohlia* sp. YN1242 and *Trentepohlia odorata* (Figure 4: oblique arrow combined with vertical arrow). (3) Two independent gains of the non-canonical code in the Trentepohliales (Figure 4: vertical arrow). The hypothesis 1 is almost impossible, since a reversal from the non-canonical to the standard genetic code is improbable due to the profound genetic changes that coincide with codon reassignment (Cocquyt et al., 2010). Several independent acquisitions of non-canonical codes have been reported for ciliates (Tourancheau et al., 1995), in the present study, the possibility that codon reassignment occurred second times independently in Trentepohliales cannot be excluded. Previous studies have shown that stop codon reassignment was a gradual process, requiring changes in the tRNA and eukaryotic release factor 1 (RF1) genes (Beier and Grimm, 2001). Some conditions are required to complete the reassignment of UGA or UAG codons to arginine codon in Trentepohliales. One is that RF1 can recognize and easily bind to the UAG/UGA glutamine codon, and the other is mutant arginine tRNA can bind to UAG or UGA codons, so that it has the ability to translate UAG or UGA into arginine. A situation has been discovered in ciliate, in addition to canonical glutamine tRNAs, two supplementary tRNAs that evolved from the normal glutamine tRNA could recognize the non-canonical TAR codons (Hanyu et al., 1986). Interestingly, there are 4 tRNA genes for arginine (*trnR-TCT*, *trnR-ACG*, *trnR-TCG*, *trnR-CCT*, and *trnR-CCT*) in all chloroplast genomes of Trentepohliales expect *Trentepohlia* sp. YN1317 and *Trentepohlia* sp. YN1242. All four tRNA genes are associated with standard arginine. Surprisingly, *trnR-TCG* and *trnR-CCT* genes are lost in *Trentepohlia* sp. YN1242 with canonical genetic code, *trnR-TCG* genes are also lost in *Trentepohlia* sp. YN1317 which has non-canonical genetic code (Supplementary Table S4). However, *Trentepohlia odorata* with canonical code had 4 tRNA for arginine like other species with non-canonical genetic code. Since the chloroplast genome of *Cephaleuros* sp. is incomplete, it is not clear how many tRNA genes for arginine are in *Cephaleuros* sp., which hinders our understanding of the non-canonical genetic code in Trentepohliales. More samples and deeper analysis of arginine tRNA is needed to clarify evolutionary scenarios in Trentepohliales.

Interestingly, a non-canonical genetic code has also been described for Trentepohliales nuclear genes, where UAG and UAA codons are reassigned to glutamine (Cocquyt et al., 2010), this result has been verified by transcriptome-based data (Li et al., 2021). The non-canonical genetic code was also found internally in chloroplast genes and nuclear genes of Cladophorales (Del Cortona et al., 2017). Considering that Cladophorales is likely to be the sister group of Trentepohliales (Cocquyt et al., 2010; Del Cortona et al., 2020), it seems reasonable to speculate that this codon reassignment occurred in the common ancestor of these two orders, prior to the drastic divergence of their chloroplast genome dynamics leading to inflated genomes in the Trentepohliales and hairpin chromosomes in the Cladophorales.

## Data Availability Statement

The datasets presented in this study can be found in online repositories. The names of the repository/repositories and accession number(s) can be found in the article/Supplementary Material.

## Author Contributions

HZ and GL designed research. JF, BL, and HZ performed research. JF and HV analyzed the data and wrote the manuscript. HZ and HV revised the draft manuscript. All authors contributed to the article and approved the submitted version.

## Conflict of Interest

The authors declare that the research was conducted in the absence of any commercial or financial relationships that could be construed as a potential conflict of interest.

## Publisher’s Note

All claims expressed in this article are solely those of the authors and do not necessarily represent those of their affiliated organizations, or those of the publisher, the editors and the reviewers. Any product that may be evaluated in this article, or claim that may be made by its manufacturer, is not guaranteed or endorsed by the publisher.

## Footnotes

1
http://sagdb.uni-goettingen.de/

2
https://megasun.bch.umontreal.ca/cgi-bin/mfannot/mfannotInterface.pl

3
https://www.ncbi.nlm.nih.gov/orffinder/

4
https://www.ncbi.nlm.nih.gov/

5
http://iqtree.cibiv.univie.ac.at/

6
http://tree.bio.ed.ac.uk/software/figtree/

## Supplementary Material

Supplementary Figure S1 Gene maps of five chloroplast genomes sequenced in this study. The gray circle on the inside shows a graph of the GC content. Arrows show the direction of transcription. Genes are color coded according to the functional categories listed in the legend at the bottom left.

Supplementary Figure S2 Maximum likelihood tree inferred from 18S rDNA sequences of Trentepohliales. Maximum likelihood bootstrap values (1000 replicates) and Bayesian posterior probabilities are given near the nodes. The algal strains studied in this paper are shaded in gray.

Supplementary Figure S3 Synteny comparison of the genus *Cephaleuros*. The colored syntenic blocks are local collinear blocks; blocks above the center line indicate they are on the same strand, and blocks below the center line indicate they are on the opposite strand.

Supplementary Figure S4 Comparison of conserved gene clusters between at least two of the six representative species of the Ulvophycean with gene order found in Trentepohliales. Black connected boxes indicate gene clusters that made up of contiguous genes. Black boxes that are contiguous but are unlinked indicate that the corresponding genes are not adjacent on the genome. Gray boxes indicate genes that are located elsewhere on genome. White boxes indicate genes that are missing from the chloroplast genomes.

Supplementary Figure S5 ML phylogenetic tree of the Chlorophyta constructed by using nucleotide data partitioned by codon position. Node support is given as maximum-likelihood (ML) bootstrap value.

Supplementary Table S1 The collection information of samples used in this study.

Supplementary Table S2 Chlorophyta taxa used in the chloroplast phylogenomic analyses.

Supplementary Table S3 Partition scheme of 31 concatenated chloroplast protein-coding genes used in this study.

Supplementary Table S4 Comparison of genes content among members of Ulvophyceae.

Supplementary Table S5 Genes with abnormal UAG/UGA codon in newly sequenced chloroplast genomes. No abnormal UAG/UGA codon was found in YN 1242 *Trentepohlia sp*. chloroplast genome.

Supplementary Table S6 Distribution and types of introns and number of intronic ORF in the newly sequenced chloroplast genomes of Trentepohliales.

## References

[B1] Altschul S. F. Madden T. L. Schaffer A. A. Zhang J. H. Zhang Z. Miller W. (1997). Gapped BLAST and PSI-BLAST: a new generation of protein database search programs. *Nucleic Acids Res.* 25 3389–3402. 10.1093/nar/25.17.3389 9254694 PMC146917

[B2] Bankevich A. Nurk S. Antipov D. Gurevich A. A. Dvorkin M. Kulikov A. S. (2012). SPAdes: a new genome assembly algorithm and its applications to single-cell sequencing. *J. Comput. Biol.* 19 455–477. 10.1089/cmb.2012.0021 22506599 PMC3342519

[B3] Beier H. Grimm M. (2001). Misreading of termination codons in eukaryotes by natural nonsense suppressor tRNAs. *Nucleic Acids Res.* 29 4767–4782. 10.1093/nar/29.23.4767 11726686 PMC96686

[B4] Bischoff H. W. Bold H. C. (1963). *Phycological Studies IV Some Soil Algae from Enchanted Rock and Related Algal Species.* Austin: University of Texas Publications. 95.

[B5] Brooks F. E. Rindi F. Suto Y. Ohtani S. Green M. (2015). The Trentepohliales (Ulvophyceae, Chlorophyta): an unusual algal order and its novel plant pathogen, Cephaleuros. *Plant Dis.* 99 740–753. 10.1094/PDIS-01-15-0029-FE 30699526

[B6] Brouard J. S. Otis C. Lemieux C. Turmel M. (2010). The exceptionally large chloroplast genome of the green alga Floydiella terrestris illuminates the evolutionary history of the Chlorophyceae. *Genome Biol. Evol.* 2 240–256. 10.1093/gbe/evq014 20624729 PMC2997540

[B7] Browne F. B. Brannen P. M. Scherm H. Taylor J. R. Shealey J. S. Fall L. A. (2019). Evaluation of disinfectants, algicides, and fungicides for control of orange cane blotch of blackberry in the field. *Crop Prot.* 122 112–117. 10.1016/j.cropro.2019.04.019

[B8] Capella-Gutierrez S. Silla-Martinez J. M. Gabaldon T. (2009). trimAl: a tool for automated alignment trimming in large-scale phylogenetic analyses. *Bioinformatics* 25 1972–1973. 10.1093/bioinformatics/btp348 19505945 PMC2712344

[B9] Chen Y. Chen Y. Shi C. Huang Z. Zhang Y. Li S. (2018). SOAPnuke: a MapReduce acceleration-supported software for integrated quality control and preprocessing of high-throughput sequencing data. *Oxford Open.* 7:1. 10.1093/gigascience/gix120 29220494 PMC5788068

[B10] Christensen A. C. (2014). Genes and junk in plant mitochondria—repair mechanisms and selection. *Genome Biol. Evol.* 6 1448–1453. 10.1093/gbe/evu115 24904012 PMC4079193

[B11] Civán̆ P. Foster P. G. Embley M. T. Séneca A. Cox C. J. (2014). Analyses of Charophyte chloroplast genomes help characterize the ancestral chloroplast genome of land plants. *Genome Biol. Evol.* 6 897–911. 10.1093/gbe/evu061 24682153 PMC4007539

[B12] Cocquyt E. Verbruggen H. Leliaert F. De Clerck O. (2010). Evolution and cytological diversification of the green seaweeds (Ulvophyceae). *Mol. Biol. Evol.* 27 2052–2061. 10.1093/molbev/msq091 20368268

[B13] Costa J. F. Lin S. M. Macaya E. C. Fernández-García C. Verbruggen H. (2016). Chloroplast genomes as a tool to resolve red algal phylogenies: a case study in the Nemaliales. *BMC Evol. Biol.* 16:205. 10.1186/s12862-016-0772-3 27724867 PMC5057469

[B14] Cremen M. C. M. Leliaert F. Marcelino V. R. Verbruggen H. (2018). Large diversity of nonstandard genes and dynamic evolution of chloroplast genomes in siphonous green algae (Bryopsidales, Chlorophyta). *Genome Biol. Evol.* 10 1048–1061. 10.1093/gbe/evy063 29635329 PMC5888179

[B15] Crooks G. E. Hon G. Chandonia J. M. Brenner S. E. (2004). WebLogo: a sequence logo generator. *Genome Res.* 14 1188–1190. 10.1101/gr.849004 15173120 PMC419797

[B16] Darling A. E. Mau B. Perna N. T. (2010). ProgressiveMauve: multiple genome alignment with gene gain, loss and rearrangement. *PLoS One* 5:e11147. 10.1371/journal.pone.0011147 20593022 PMC2892488

[B17] Del Cortona A. Jackson C. J. Bucchini F. Van Bel M. D’hondt S. Škaloud P. (2020). Neoproterozoic origin and multiple transitions to macroscopic growth in green seaweeds. *PNAS* 117:2551. 10.1073/pnas.1910060117 31911467 PMC7007542

[B18] Del Cortona A. Leliaert F. Bogaert K. A. Turmel M. Boedeker C. Janouškovec J. (2017). The plastid genome in Cladophorales green algae is encoded by hairpin Chromosomes. *Curr. Biol.* 27 3771–3782. 10.1016/j.cub.2017.11.004 29199074

[B19] Fang J. Li S. Liu B. Liu G. Hu Z. Zhu H. (2021). Molecular phylogeny and morphology of Cephaleuros (Trentepohliales, Chlorophyta) from southern China. *Phycologia* 2021 1–11. 10.1080/00318884.2021.1884799

[B20] Fang L. Leliaert F. Novis P. M. Zhang Z. H. Zhu H. Liu G. X. (2018). Improving phylogenetic inference of core Chlorophyta using chloroplast sequences with strong phylogenetic signals and heterogeneous models. *Mol. Phylogenet. Evol.* 127 248–255. 10.1016/j.ympev.2018.06.006 29885933

[B21] Fučíková K. Leliaert F. Cooper E. D. Skaloud P. D’Hondt S. De Clerck O. (2014). New phylogenetic hypotheses for the core Chlorophyta based on chloroplast sequence data. *Front. Ecol. Evol.* 2:63. 10.3389/fevo.2014.00063

[B22] Gulbrandsen ØS. Andresen I. J. Krabberød A. K. Brate J. Shalchian-Tabrizi K. (2021). Phylogenomic analysis restructures the ulvophyceae. *J. Phycol.* 57 1223–1233. 10.1111/jpy.13168-20-145 33721355

[B23] Hanyu N. Kuchino Y. Nishimura S. Beier H. (1986). Dramatic events in ciliate evolution: alteration of UAA and UAG termination codons to glutamine codons due to anticodon mutations in two Tetrahymena tRNAsGln. *Embo J.* 5 1307–1311. 16453685 10.1002/j.1460-2075.1986.tb04360.x PMC1166941

[B24] Huelsenbeck J. P. Ronquist F. (2001). MRBAYES: bayesian inference of phylogenetic trees. *Bioinformatics* 17 754–755. 10.1093/bioinformatics/17.8.754 11524383

[B25] Jackson C. Knoll A. H. Chan C. X. Verbruggen H. (2018). Plastid phylogenomics with broad taxon sampling further elucidates the distinct evolutionary origins and timing of secondary green plastids. *Sci. Rep.* 8:1523. 10.1038/s41598-017-18805-w 29367699 PMC5784168

[B26] Kalyaanamoorthy S. Minh B. Q. Wong T. Haeseler A. V. Jermiin L. S. (2017). ModelFinder: fast Model Selection for Accurate Phylogenetic Estimates. *Nat. Methods.* 14 587–589. 10.1038/nmeth.4285 28481363 PMC5453245

[B27] Karin L. Peter H. Andreas R. E. Stærfeldt H. H. Torbjørn R. Ussery D. W. (2007). RNAmmer: consistent and rapid annotation of ribosomal RNA genes. *Nucleic Acids Res.* 35 3100–3108. 10.1093/nar/gkm160 17452365 PMC1888812

[B28] Katoh K. Standley D. M. (2014). MAFFT: iterative refinement and additional methods. *Mol Biol Evol.* 1079:131. 10.1007/978-1-62703-646-7_8 24170399

[B29] Kosecka M. Jablonska A. Flakus A. Rodriguez-Flakus P. Kukwa M. Guzow-Krzeminska B. (2020). Trentepohlialean Algae (Trentepohliales, Ulvophyceae) Show preference to selected mycobiont lineages in lichen symbioses. *J. Phycol.* 56 979–993. 10.1111/jpy.12994 32198895

[B30] Lang-Unnasch N. Aiello D. P. (1999). Sequence evidence for an altered genetic code in the Neospora caninum plastid. *Int. J. Parasit.* 29 1557–1562. 10.1016/s0020-7519(99)00119-8 10608442

[B31] Lee S. H. Lin S. R. Chen S. F. (2020). Identification of tea foliar diseases and pest damage under practical field conditions using convolutional neural network. *Plant Pathol.* 69 1731–1739. 10.1111/ppa.13251

[B32] Leliaert F. Lopez-Bautista J. M. (2015). The chloroplast genomes of Bryopsis plumosa and Tydemania expeditionis (Bryopsidales, Chlorophyta): compact genomes and genes of bacterial origin. *BMC Genomics* 16:204. 10.1186/s12864-015-1418-3 25879186 PMC4487195

[B33] Lemieux C. Otis C. Turmel M. (2014). Chloroplast phylogenomic analysis resolves deep-level relationships within the green algal class Trebouxiophyceae. *BMC Evol. Biol.* 14:211. 10.1186/s12862-014-0211-2 25270575 PMC4189289

[B34] Li Q. Liu J. Zhang L. Liu Q. (2014). De novo transcriptome analysis of an aerial microalga Trentepohlia jolithus: pathway description and gene discovery for carbon fixation and carotenoid biosynthesis. *PLoS One* 9:e108488. 10.1371/journal.pone.0108488 25254555 PMC4177907

[B35] Li X. Hou Z. Xu C. Shi X. Yang L. Lewis L. A. (2021). Large phylogenomic datasets reveal deep relationships and trait evolution in chlorophyte green algae. *Genome Biol. Evol.* 13:evab101. 33950183 10.1093/gbe/evab101 PMC8271138

[B36] Liu G. X. Zhang Q. Zhu H. Hu Z. Y. (2012). Massive Trentepohlia-Bloom in a Glacier Valley of Mt. Gongga, China, and a New Variety of Trentepohlia (Chlorophyta). *PLoS One* 7:e37725. 10.1371/journal.pone.0037725 22815686 PMC3398038

[B37] López-Bautista J. M. Rindi F. Guiry M. D. (2006). Molecular systematics of the subaerial green algal order Trentepohliales: an assessment based on morphological and molecular data. *Int. J. Syst. Evol. Microbiol.* 56 1709–1715. 10.1016/j.phymed.2008.10.009 16825655

[B38] Lowe T. M. Eddy S. R. (1997). tRNAscan-SE: a program for improved detection of transfer RNA genes in genomic sequence. *Nucleic Acids Res.* 25 955–964. 10.1093/nar/25.5.0955 9023104 PMC146525

[B39] Lynch M. (2007). *The Origins of Genome Architecture*. New York, NY: Oxford University Press.

[B40] Lynch M. Koskella B. Schaack S. (2006). Mutation pressure and the evolution of organelle genomic architecture. *Science* 311 1727–1730. 10.1126/science.1118884 16556832

[B41] Matsumoto T. Ishikawa S. A. Hashimoto T. Inagaki Y. (2011). A deviant genetic code in the green alga-derived plastid in the dinoflagellate Lepidodinium chlorophorum. *Mol. Phylogenet. Evol.* 60 68–72. 10.1016/j.ympev.2011.04.010 21530665

[B42] Melton J. T. Leliaert F. Tronholm A. Lopez-Bautista J. M. (2015). The complete chloroplast and mitochondrial Genomes of the green macroalga Ulva sp UNA00071828 (Ulvophyceae, Chlorophyta). *PLoS One* 10:e0121020. 10.1371/journal.pone.0121020 25849557 PMC4388391

[B43] Michel F. Umesono K. Ozeki H. (1989). Comparative and functional anatomy of group II catalytic introns–a review. *Gene* 82 5–30. 10.1016/0378-1119(89)90026-7 2684776

[B44] Michel F. Westhof E. (1990). Modelling of the three-dimensional architecture of group I catalytic introns based on comparative sequence analysis. *J. Mol. Biol.* 216 585–610. 10.1016/0022-2836(90)90386-Z 2258934

[B45] Nelsen M. P. Plata E. R. Andrew C. J. Lücking R. Lumbsch H. T. (2011). Phylogenetic diversity of Trentepohlialean algae associated with lichen-forming fungi. *J. Phycol.* 47 282–290. 10.1111/j.1529-8817.2011.00962.x 27021860

[B46] Nelson S. C. (2008). *Cephaleuros Species, the Plant-parasitic Green Algae.* Hawaii: University of Hawaii. 43.

[B47] Pasella M. M. Lee M.-F. E. Marcelino V. R. Willis A. Verbruggen H. (2021). Ten Ostreobium (Ulvophyceae) strains from great barrier reef corals as a resource for algal endolith biology and genomics. *Biorxiv* 10.1101/2021.08.16.453452

[B48] Pombert J. F. Otis C. Lemieux C. Turmel M. (2005). Chloroplast genome sequence of the green alga Pseudendoclonium akinetum (Ulvophyceae) reveals unusual structural features and new insights into the branching order of chlorophyte lineages. *Mol. Biol. Evol.* 22 1903–1918. 10.1093/molbev/msi182 15930151

[B49] Qu X. J. Moore M. J. Li D. Z. Yi T. S. (2019). PGA: a software package for rapid, accurate, and flexible batch annotation of plastomes. *Plant Methods* 15 1–12. 10.1186/s13007-019-0435-7 31139240 PMC6528300

[B50] R Core Team. (2016). *R**: a Language and Environment for Statistical Computing. R Foundation for Statistical Computing.* Vienna. Available online at: URL. https://www.R-project.org/ (accessed August 15, 2021).

[B51] Repetti S. I. Jackson C. J. Judd L. M. Wick R. R. Holt K. E. Verbruggen H. (2020). The inflated mitochondrial genomes of siphonous green algae reflect processes driving expansion of noncoding DNA and proliferation of introns. *PeerJ.* 8:e8273. 10.7717/peerj.8273 31915577 PMC6944098

[B52] Rindi F. Lam D. W. López-Bautista J. M. (2009). Phylogenetic relationships and species circumscription in Trentepohlia and Printzina (Trentepohliales, Chlorophyta). *Mol. Phylogenet. Evol.* 52 329–339. 10.1016/j.ympev.2009.01.009 19489121

[B53] Smith D. R. (2016). The mutational hazard hypothesis of organelle genome evolution: 10 years on. *Mol. Ecol.* 25 3769–3775. 10.1111/mec.13742 27357487

[B54] Stephan G. Pascal L. Ralph B. (2019). OrganellarGenomeDRAW (OGDRAW) version 1.3.1: expanded toolkit for the graphical visualization of organellar genomes. *Nucleic Acids Res.* 47 W59–W64. 10.1101/545509 30949694 PMC6602502

[B55] Sun L. Fang L. Zhang Z. Chang X. Penny D. Zhong B. (2016). Chloroplast phylogenomic inference of green algae relationships. *Sci. Rep.* 6:20528. 10.1038/srep20528 26846729 PMC4742797

[B56] Suto Y. Ohtani S. (2009). Morphology and taxonomy of five Cephaleuros species (Trentepohliaceae, Chlorophyta) from Japan, including three new species. *Phycologia* 48 213–236. 10.2216/07-31.1

[B57] Talavera G. Castresana J. (2007). Improvement of phylogenies after removing divergent and ambiguously aligned blocks from protein sequence alignments. *Syst. Biol.* 56 564–577. 10.1080/10635150701472164 17654362

[B58] Tamura K. Dudley J. Nei M. Kumar S. (2007). MEGA4: molecular Evolutionary Genetics Analysis (MEGA) software version 4.0. *Mol. Biol. Evol.* 24 1596–1599. 10.1093/molbev/msm092 17488738

[B59] Thompson R. H. Wujek D. E. (1997). *Trentepohliales: cephaleuros, Phycopeltis, and Stomatochroon : morphology, Taxonomy, and Ecology Science Publishers Inc., PO Box 699, Enfield, New Hampshire 03748, USA, 1997, $93. 149 pp.* Cambridge: Cambridge University Press.

[B60] Tourancheau A. B. Tsao N. Klobutcher L. A. Pearlman R. E. Adoutte A. (1995). Genetic-code deviations in the ciliates - evidence for multiple and independent events. *Embo J.* 14 3262–3267. 10.1002/j.1460-2075.1995.tb07329.x 7621837 PMC394388

[B61] Trifinopoulos J. Nguyen L. T. von Haeseler A. Minh B. Q. (2016). W-IQ-TREE: a fast online phylogenetic tool for maximum likelihood analysis. *Nucleic Acids Res.* 44 W232–W235. 10.1093/nar/gkw256 27084950 PMC4987875

[B62] Turmel M. Lemieux C. (2017). Evolution of the plastid genome in green algae. *Adv. Bot. Res.* 85, 157–193. 10.1016/bs.abr.2017.11.010

[B63] Turmel M. Otis C. Lemieux C. (2009). The Chloroplast genomes of the green algae Pedinomonas minor, Parachlorella kessleri, and Oocystis solitatia reveal a shared ancestry between the Pedinomonadales and Chlorellales. *Mol. Biol. Evol.* 26 2317–2331. 10.1093/molbev/msp138 19578159

[B64] Turmel M. Otis C. Lemieux C. (2015). Dynamic Evolution of the Chloroplast Genome in the Green Algal Classes Pedinophyceae and Trebouxiophyceae. *Genome Biol. Evol.* 7 2062–2082. 10.1093/gbe/evv130 26139832 PMC4524492

[B65] Turmel M. Otis C. Lemieux C. (2017). Divergent copies of the large inverted repeat in the chloroplast genomes of ulvophycean green algae. *Sci. Rep.* 7:994. 10.1038/s41598-017-01144-1 28428552 PMC5430533

[B66] Verbruggen H. Marcelino V. R. Guiry M. D. Cremen M. C. M. Jackson C. J. (2017). Phylogenetic position of the coral symbiont Ostreobium (Ulvophyceae) inferred from chloroplast genome data. *J. Phycol.* 53 790–803. 10.1111/jpy.12540 28394415

[B67] Wolf F. A. (1930). A parasitic alga, Cephaleuros virescens Kunze, on citrus and certain other plants. *J. Elisha Mitchell Sci. Soc.* 45 187–205.

[B68] Xu J. Liu C. Song Y. Li M. (2021). Comparative analysis of the chloroplast genome for four Pennisetum Species: molecular structure and phylogenetic relationships. *Front. Genet.* 12:1248. 10.3389/fgene.2021.687844 34386040 PMC8354216

[B69] Zhang D. Gao F. Jakovlić I. Zou H. Zhang J. Li W. X. (2020). PhyloSuite: an integrated and scalable desktop platform for streamlined molecular sequence data management and evolutionary phylogenetics studies. *Mol. Ecol. Resour.* 20 348–355. 10.1111/1755-0998.13096 31599058

[B70] Zheng F. Wang B. Shen Z. Wang Z. Wang W. Liu H. (2020). The chloroplast genome sequence of the green macroalga Caulerpa okamurae (Ulvophyceae, Chlorophyta): its structural features, organization and phylogenetic analysis. *Mar. Genom.* 53:100752. 10.1016/j.margen.2020.100752 32014385

[B71] Zhu H. Hu Y. X. Liu F. Hu Z. Y. Liu G. X. (2019). Characterization of the Chloroplast Genome of Trentepohlia odorata (Trentepohliales, Chlorophyta), and discussion of its taxonomy. *Int. J. Mol. Sci.* 20:1774. 10.3390/ijms20071774 30974837 PMC6480257

[B72] Zhu H. Hu Z. Y. Liu G. X. (2017). Morphology and molecular phylogeny of Trentepohliales (Chlorophyta) from China. *Eur. J. Phycol.* 52 330–341. 10.1080/09670262.2017.1309574

