# Supplementary figures and images for: Six Newly Sequenced Chloroplast Genomes From Trentepohliales: The Inflated Genomes, Alternative Genetic Code and Dynamic Evolution
Source: Front Plant Sci. 2021 Dec 8;12:780054. doi: 10.3389/fpls.2021.780054 (PMC8692980; doi:10.3389/fpls.2021.780054)

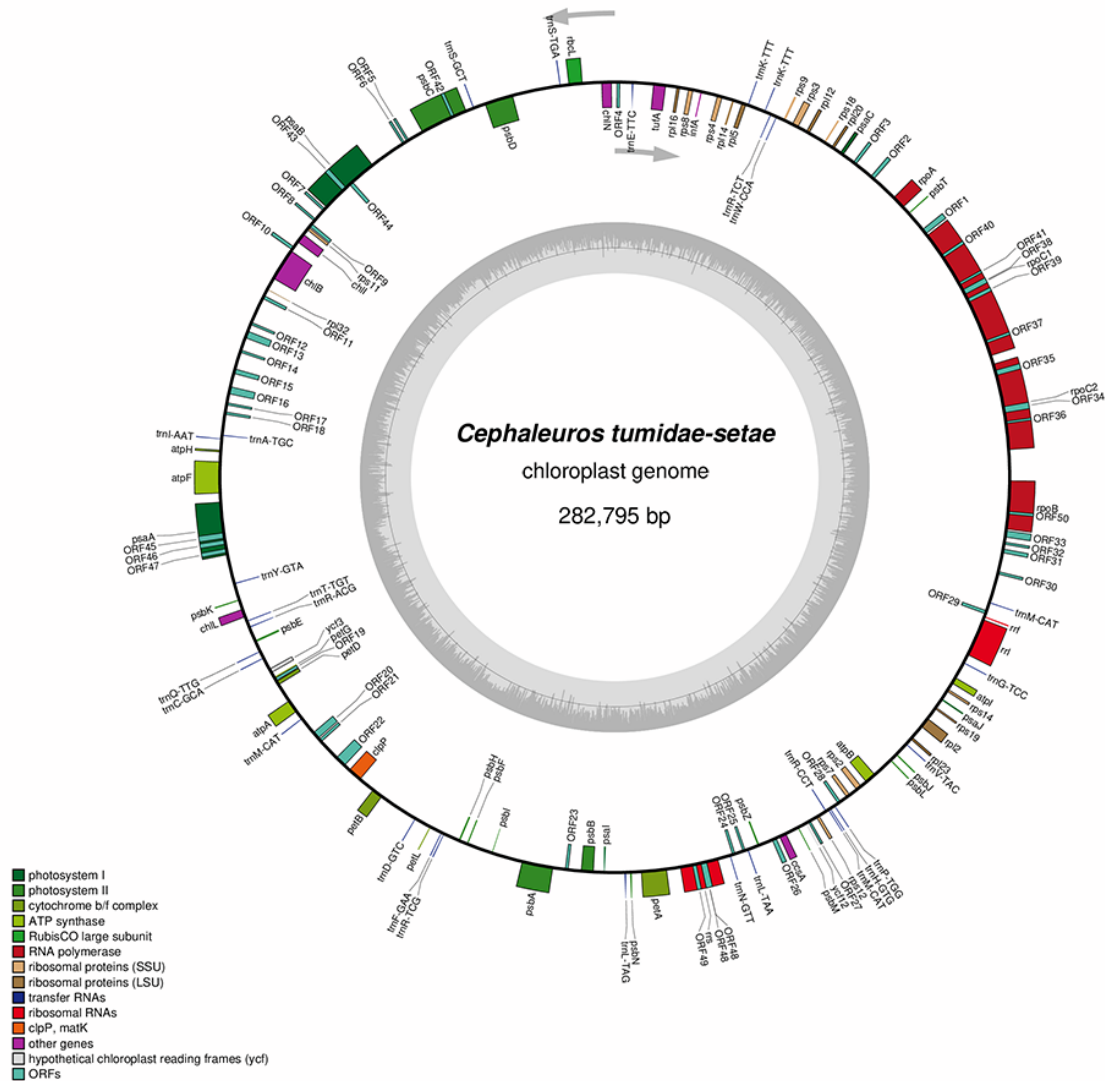

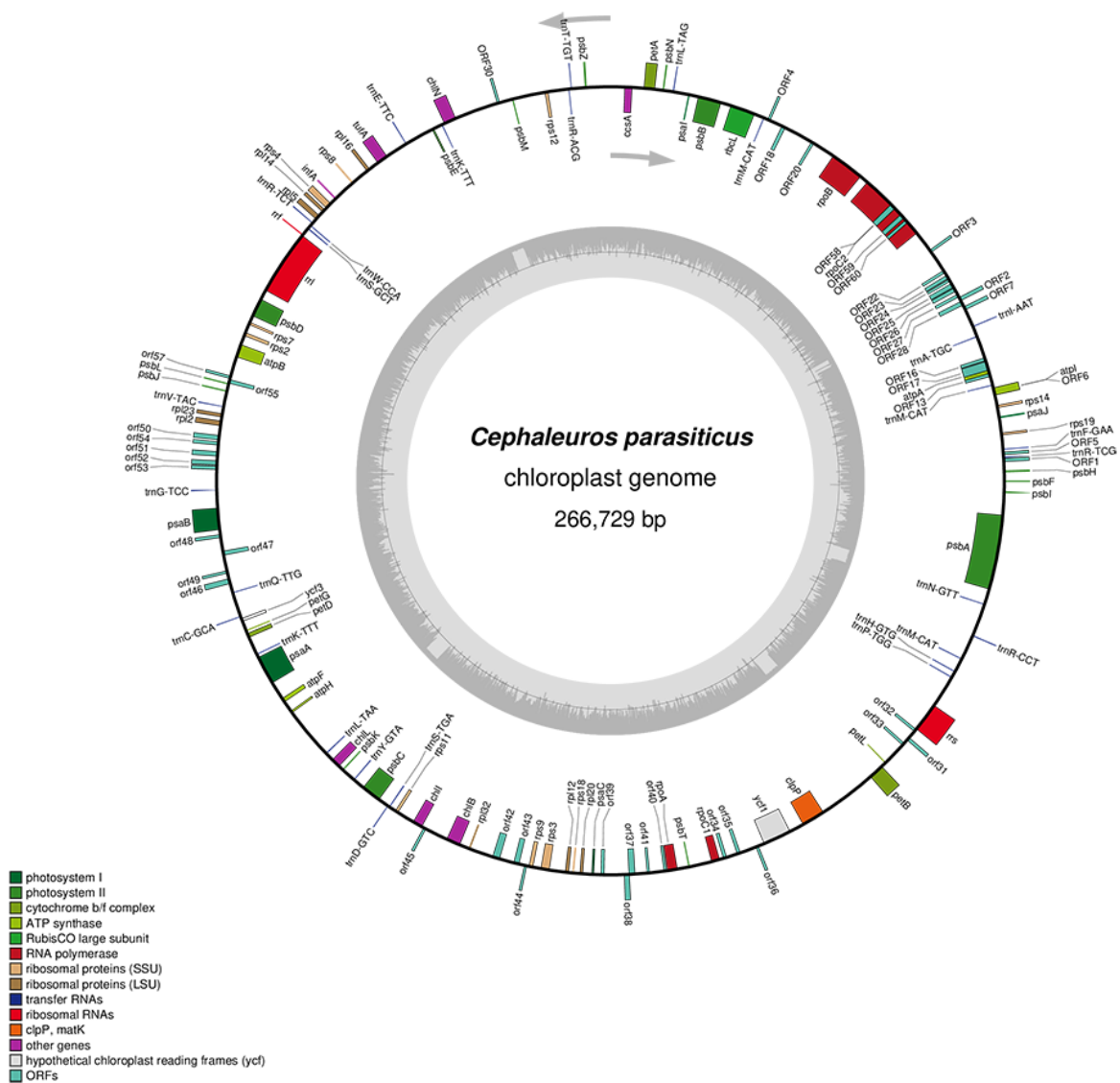

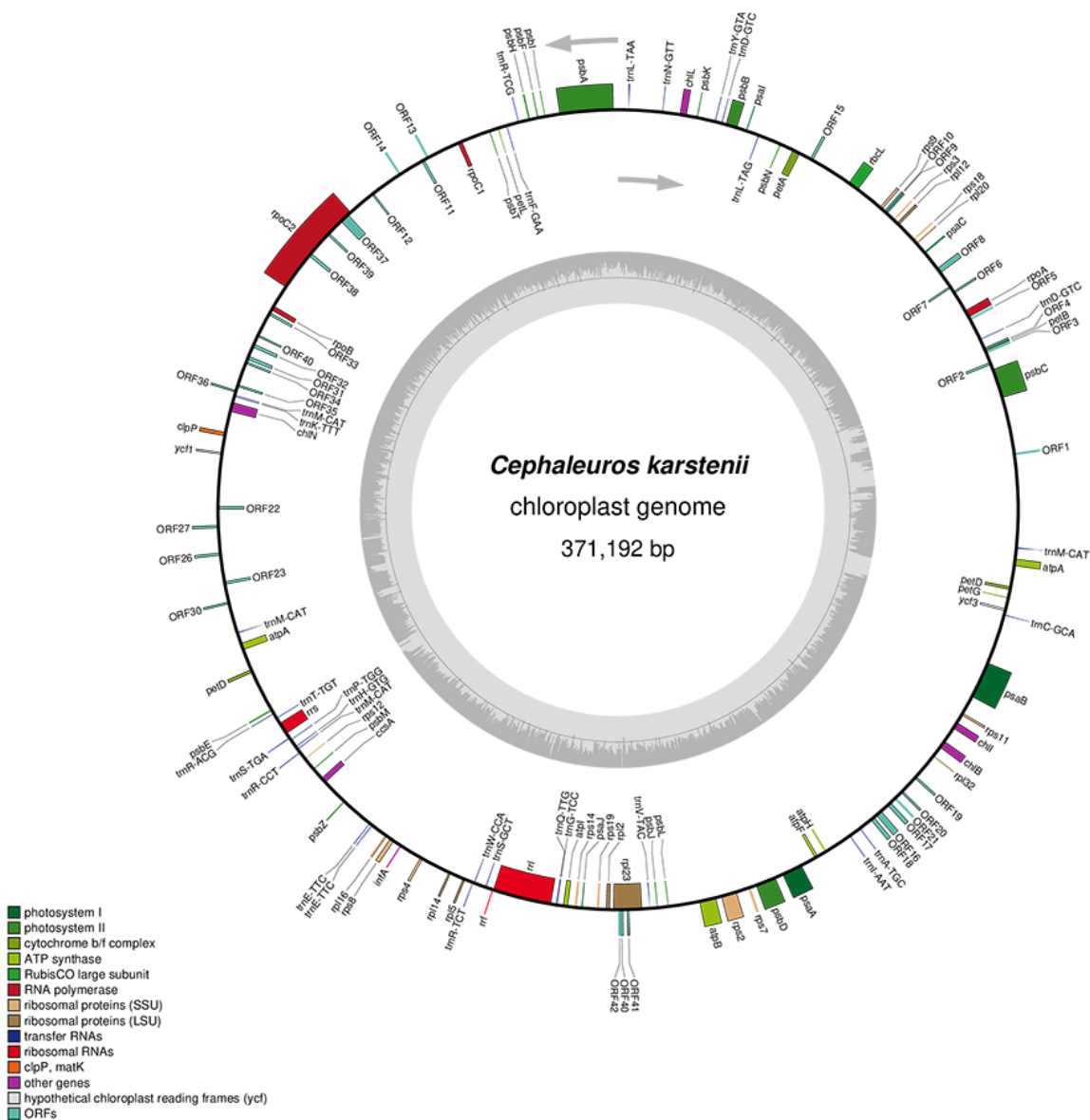

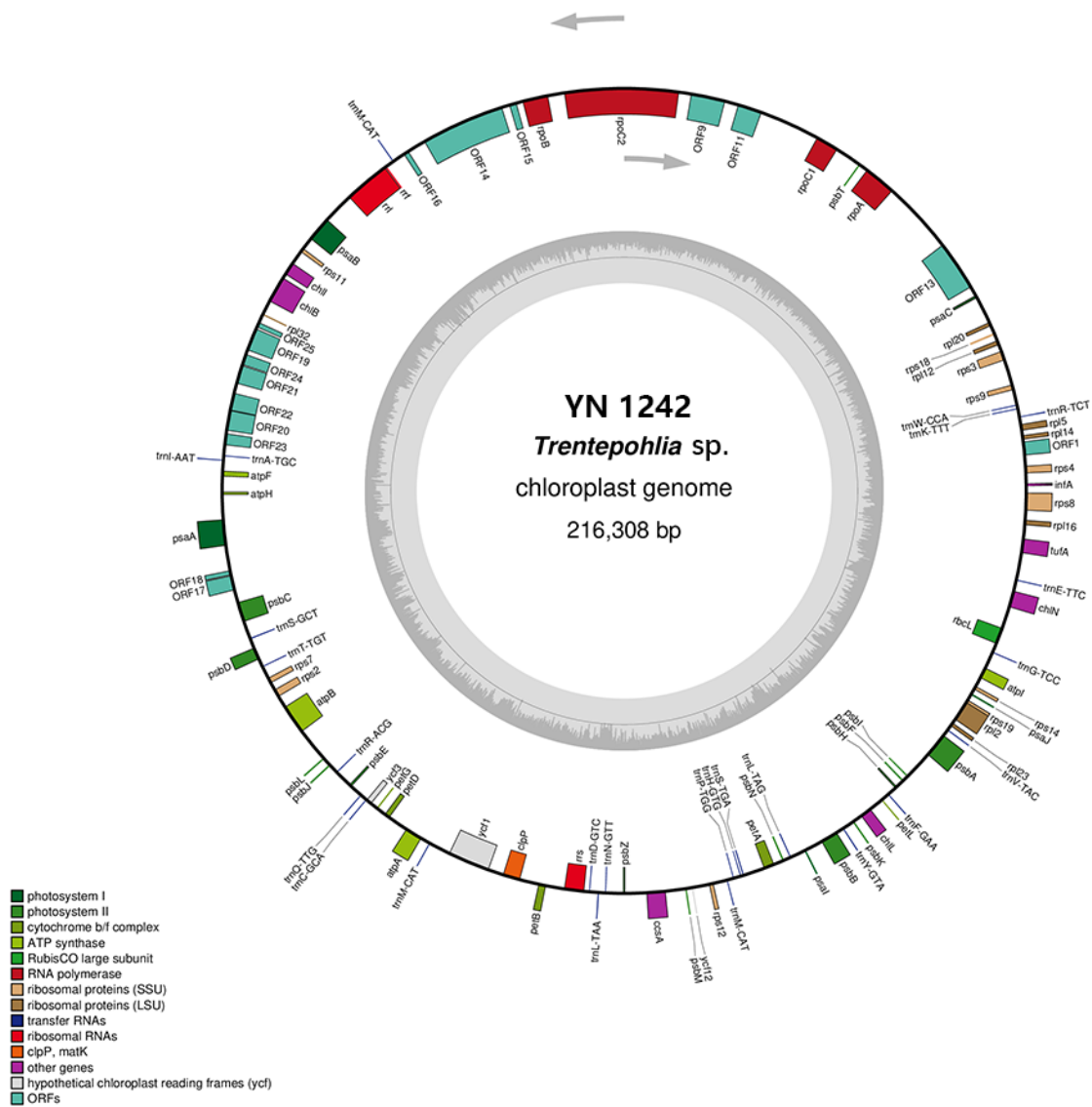

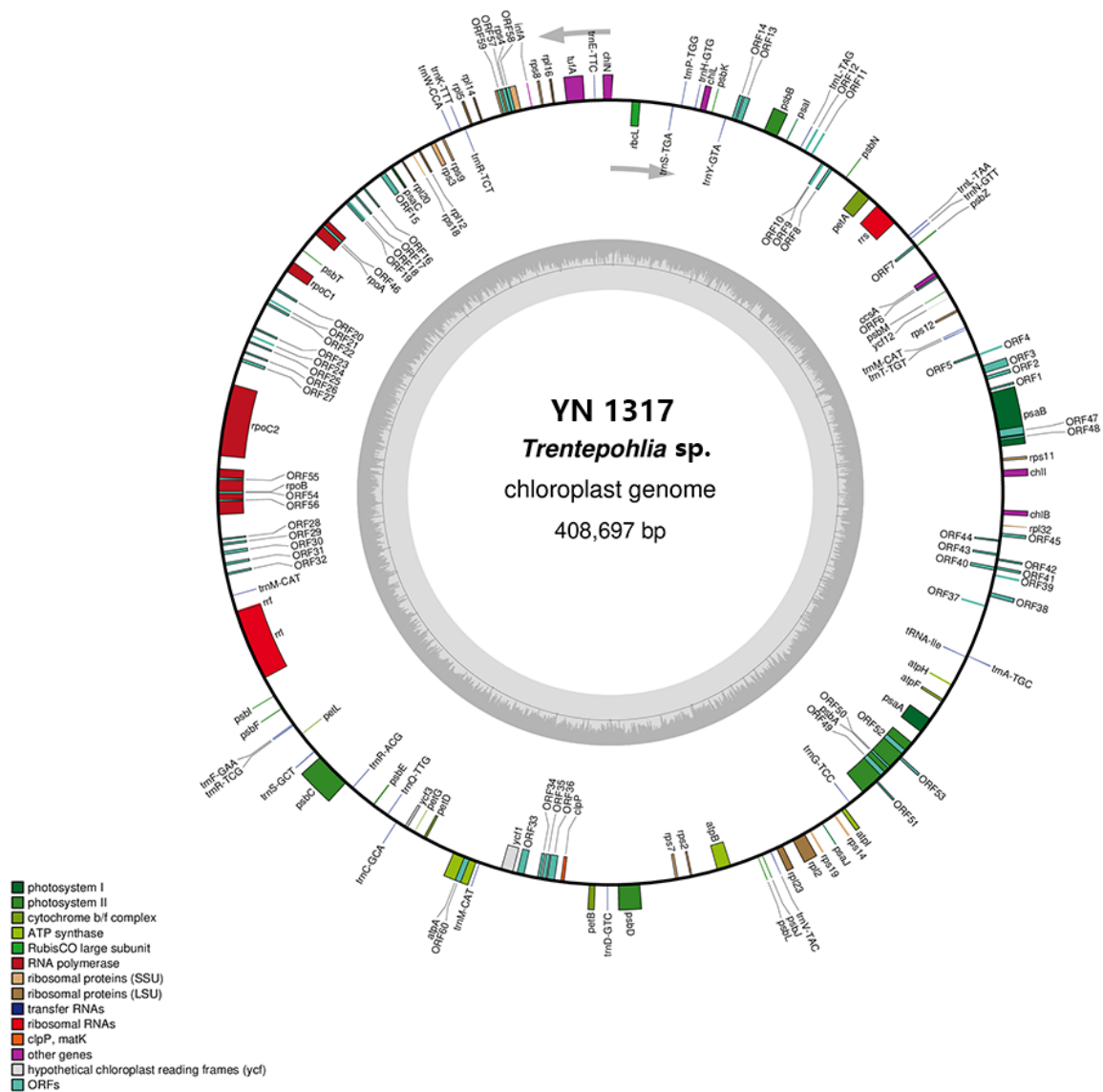

Supplement: Supplementary Figure S1 — Gene maps of five chloroplast genomes sequenced in this study. The gray circle on the inside shows a graph of the GC content. Arrows show the direction of transcription. Genes are color coded according to the functional categories listed in the legend at the bottom left. [file Data_Sheet_1.PDF]
