# Supplementary material for: Six Newly Sequenced Chloroplast Genomes From Trentepohliales: The Inflated Genomes, Alternative Genetic Code and Dynamic Evolution
Source: Front Plant Sci. 2021 Dec 8;12:780054. doi: 10.3389/fpls.2021.780054 (PMC8692980; doi:10.3389/fpls.2021.780054)
Supplement: Supplementary Table S1 — The collection information of samples used in this study. [file Table_1.docx]

Supplementary Table S1. The collection information of strains used in this study.

| Species | Strain number | Collection date | Sampling Location | Isolate sources | Origin | References |
| --- | --- | --- | --- | --- | --- | --- |
| *Cephaleuros virescens* | SAG 42.85 | 1985.09 | Taiwan | Surface of leaves | Filaments | Rindi et al., 2017 |
| *Cephaleuros* *tumidae*-*setae* | BN 17 | 2018.08.30 | Xishuangbanna Tropical Botanical Garden in Yunnan province, China | Surface of leaves | Filaments | Fang et al., 2021 |
| *Cephaleuros karstenni* | GD1942 | 2019.04.10 | South China Botanical Garden in Guangdong province, China | Surface of leaves | Filaments | Fang et al., 2021 |
| *Cephaleuros parasiticus* | GD1927 | 2019.04.10 | South China Botanical Garden in Guangdong province, China | Surface of leaves | Filaments | Fang et al., 2021 |
| *Trentepohlia* sp. | YN1242 | 2012.06.22 | Xishuangbanna Tropical Botanical Garden in Yunnan province, China | Surface of iron pipe | Filaments | Zhu et al., 2017 |
| *Trentepohlia* sp. | YN1317 | 2013.05.13 | Xishuangbanna Tropical Botanical Garden in Yunnan province, China | Surface of trunk | Filaments | Zhu et al., 2017 |
