# Supplementary material for: Six Newly Sequenced Chloroplast Genomes From Trentepohliales: The Inflated Genomes, Alternative Genetic Code and Dynamic Evolution
Source: Front Plant Sci. 2021 Dec 8;12:780054. doi: 10.3389/fpls.2021.780054 (PMC8692980; doi:10.3389/fpls.2021.780054)
Supplement: Supplementary Table S3 — Partition scheme of 31 concatenated chloroplast protein-coding genes used in this study. [file Table_3.docx]

| Subset | Best model | Partition scheme |
| --- | --- | --- |
| 1 | GTR+F+I+G4 | *atp*A, *atp*B, *atp*H, *clp*P, *pet*B, *pet*D, *psa*B, *psa*C, *psb*B, *rbc*L, *rpl*14, *rpl*2, *rpl*5, *rps*11, *rps*7, *rps*8, t*uf*A, *ycf*3, *psb*M, *psb*Z |
| 2 | TVM+F+I+G4 | *atp*F, *psb*K, *psb*N,, *rps*9, *pet*G, *rpl*20 |
| 3 | GTR+F+G4 | *psb*J, *psb*A, |
| 4 | TPM2u+F+I+G4 | *rpl*16 |
| 5 | TPM3u+F+I+G4 | *rps*18, *rps*19 |

**Supplementary Table S3.** Partition scheme of 31 concatenated chloroplast protein-coding genes used in this study.
